# Improving Access to Mental Health Services for Korean American Immigrants: Moving Toward a Community Partnership Between Religious and Mental Health Services

**DOI:** 10.4306/pi.2008.5.1.14

**Published:** 2008-03-31

**Authors:** Hochang B. Lee, Jennifer A. Hanner, Seong-Jin Cho, Hae-Ra Han, Miyong T. Kim

**Affiliations:** 1 Department of Psychiatry and Behavioral Sciences, School of Medicine, Johns Hopkins University, Baltimore, Maryland, US.; 2 Department of Psychiatry, Gachon Unversity of Medicine and Science Gil Medical Center, Incheon, Korea.; 3 School of Nursing, Johns Hopkins University, Baltimore, Maryland, US.

**Keywords:** Korean, Korean American, Immigration, Mental health services, Disarity, Religion, Church

## Abstract

Korean Americans (KAs) with psychiatric service needs underutilizes the mainstream mental health services in United States (US). Barriers to mental health service access among KAs reflect their unique heritage and culture. More than two-thirds of KAs identify themselves as Christians, and Korean clergy have influential roles in daily lives of vast majority of KAs. By working with the Korean clergy, a small voluntary organization such as the Association of Korean American Psychiatrists could provide invaluable assistance in removing the barriers to mental health services for KAs.

## Characteristics of the Korean American Population

Korean Americans (KAs) are one of the most homogeneous Asian populations in terms of race, language, and other cultural factors. They are also one of the fastest-growing Asian sub-populations in the United States (US). According to the 2000 Census, KAs comprise 11% of the total Asian-American population in US, numbering approximately 1.1 million people.^1^ Approximately 40% of Koreans live in the Southern California metropolitan region (Los Angeles-Riverside-Orange-San-Bernadino-Ventura country area), as well as the metropolitan areas of New York, northern New Jersey, Connecticut and Pennsylvania.^2^ It is estimated that 96% of Korean immigrants live in or near metropolitan areas, and the majority (57%) of them live in the suburbs.^2^ Compared to other Asian minority ethnic groups (e.g. Chinese Americans), KAs are more widely dispersed geographically.^2^

Approximately two-thirds of KAs are "foreign-born" or first-generation immigrants, and their Korean culture and heritage remain important in their daily lives.^1^ Many of them immigrated between 1970s and 1980s, after US immigration laws were liberalized in 1960's. A typical pattern for a newly arriving Korean family has been to start a small business with capital either brought from Korea or saved from a few years of manual blue-collar labor. More than one-third of Korean immigrant households operate in self-owned businesses that are small in scale, labor-intensive, and family or individually operated while another one-fifth engage in professional work, and the rest in other salaried occupations.^3^ A unique aspect of the KA population is the high proportion (more than two-thirds) who identify themselves as Christians.^4^ Of these, 60-65% identify themselves as Protestants and 10-15% as Roman Catholics. As the centers of religious, political, social, and educational activities for KAs, Korean churches have a profound impact in on daily lives of KAs.^4^

## Health Service Utilization by Korean Americans in United States

Despite the rapid increase in the size of the KA population, the status of KAs in the US healthcare system has remained marginal. Data regarding patterns of underutilization of general health services by KAs. For example, when compared to other ethnic minority groups, KAs rank among the lowest in having medical insurance (42% without health insurance coverage, whether private, government-sponsored, Medicare/Medicaid).^5^ This lack of health insurance coverage is likely related to their recent immigration history and reliance on income from small retail businesses, which does not allow them to afford health insurance premiums. KAs ranks the lowest among ethnic minority groups in terms of their utilization of health care services, most likely, because of their lack of health insurance and their limited English speaking skills; only 51% get a regular annual check-up by their primary physician.^6^ With their high incidence and risk of smoking, alcohol use, hypertension, diabetes and liver disease, KAs are in critical need of culturally sensitive and systematic interventions to encourage healthy behavior and provide assistance in managing chronic illnesses.^7^

## Prevalence of Mental Disorders Among Korean Americans

Most of the epidemiological data regarding the prevalence of mental disorders among Asian Americans is specific to Chinese-Americans. One of the earliest community studies of depression and Asian Americans, conducted in the 1970's, found that 40% of Chinese Americans had feelings of depression.^8^ More recently, the Chinese American Psychiatric Epidemiologic Study found a lifetime prevalence rate for major depression of 6.9% and a twelve-month rate of 3.4%.^9^ Interestingly, using a similar diagnostic instrument, the National Co-morbidity Study reported vastly higher rates of major depression in general population at a 17% lifetime and 10% current rate.^10^ Even more recently, the National Latino and Asian American Study (NLAAS) conducted the first national epidemiological survey of 2095 Asian Americans (Chinese, Filipino, Vietnamese, and other Asians including Korean Americans) in the United States and reported that overall lifetime rate of any mental disorder to be 17.30% and the 12 month rate was 9.19%.^11^ Again, these figures are much lower than the 12-month prevalence of 26.2% and lifetime prevalence of 46.4% for any psychiatric disorders of the Diagnostic and Statistical Manual of Mental Disorders, fourth edition (DSM-IV)^12^ in the general population reported by the National Co-morbidity Survey Replication.^13^,^14^ No clear explanation has been offered for the conflicting findings between these two studies, but the discrepancy attests to the complexity of diagnosing mental disorders in Asian-American subjects who are more likely to express their problems in behavioral or somatic terms rather than emotional ones. At present, we await the prevalence data for specific DSM-IV psychiatric disorders from the NLAAS.

The prevalence of mental illness among KAs is poorly documented. The few available community-based epidemiological studies have reported that clinical depression is at least as prevalent as it is in the general population. A Seattle-based study made similar observations for Chinese, Filipino, Japanese, and Korean immigrants, who all reported a higher number of depressive symptoms than did white Americans.^8^ The same study found that Korean Americans had the highest depression symptom scores, followed by Filipino, Japanese, and Chinese Americans. Hurh and coworkers conducted diagnostic interviews of 622 Korean immigrants (20 years of age and older) residing in the Chicago area and found that the mean Center for Epidemiological Studies-Depression Scale (CES-D) scores (12.3 for men and 12.9 for women) among KAs was three to four points higher than those of white Americans and other Asian-Americans.^15^ Noh and colleagues also utilized the CES-D to measure depression by applying DSM-III criteria and compared the prevalence among Korean immigrants and the larger communities in Canada and the US.^16^ They reported that depressive syndrome was present in 4.5% of the sample, a rate similar to that reported by the Epidemiologic Catchment Area studies. Only one study is available regarding the prevalence of psychiatric disorders among elderly Koreans in the US. Yamamoto and coworkers examined the lifetime prevalence of various psychiatric disorders among 100 Korean elderly in Los Angeles, based on the Diagnostic Interview Schedule and found a comparable prevalence of psychiatric symptoms (including depression) among KA and Caucasian seniors in St. Louis and in Korean seniors in Korea.^17^

Few published studies has examined correlates of depression among KAs. The results of this study indicated that being single or divorced, having a lower level of education, and unemployment were associated with depressive symptoms.^15^ The same study also reported that significant gender differences in the correlates were observed. In the case of KA men, a set of work-related variables were the strongest correlates of KA men's mental health, whereas family life satisfaction and ethnic attachment variables were moderately associated with KA women. Similar findings have been reported for correlates of depression among Korean immigrants in Canada.^18^

## Mental Health Service Utilization by Korean Americans

Specific data on mental health service utilization among KAs are also scarce. Nevertheless, a relative lack of utilization of mental health services among KAs can be inferred from previous reports of an Asian American trend toward underutilization of mental health services. Extremely low admission rates to state hospitals have been consistently documented for Asians in the past.^19^ Based on the review of National trends in minority utilization of mental health services, Cheung have reported that Asian American/Pacific Islanders use both inpatient and outpatient mental health services less often than whites and African-Americans.^20^ These findings have been augmented by equally substantive reports of their low utilization of outpatient mental health services.^21^ Previously, Sue and Morishima have investigated the services received, length of treatment, and outcomes of thousands of clients using outpatient services in the Los Angeles County Mental Health system.^22^ They reported that compared to Caucasian and African-Americans, the Asian Americans and Mexican Americans underutilized services. More recently, the results from the NLAAS suggest that Asian Americans have lower rates of mental health-related service use compared with the general population; only 8.6% of Asian Americans sought help from any services versus 17.9% of the general population in the National Co-morbidity Survey Replication Study.^23^ This low level of service use may be accounted for by a lack of knowledge of existing resources and unfamiliarity with treatment methods as well as the failure of mental health services to provide culturally relevant interventions. One published report, derived mainly from treatment sources, has suggested that KAs have had comparatively lower admission rates to mental health facilities and a lower use of mental health services than other ethnic groups.^24^ Combined with the comparable prevalence of psychiatric disorders among KAs and in the general population, this trend toward mental health service underutilization among Asian-Americans strongly implies unmet treatment needs among KAs as well.

## Barriers to Access to Mental Health Services Among Korean Americans

For KAs, the barriers to access to mainstream mental health services are similar to those for other Asian ethnic groups in the US. Among Asian immigrants, the frequently cited barriers to adequate mental health care are: 1) inability to speak English or lack of an available interpreter, 2) an inadequate number of trained mental health workers, especially psychiatrists who are culturally sensitive, 3) stigmas associated with mental disorders, 4) limited awareness of mental health disorders within the community, 5) a lack of awareness about available mental health services, and 6) belief in ethnic traditional medicine. In particular, the language issue is a common barrier to service across the Asian American ethnic groups, and the Surgeon General's Report on Mental Health states that nearly half of the Asian American/Pacific Islander population's low utilization of mental health service is attributable to s lack of English proficiency and a shortage of providers who possess appropriate language skills.^25^

Within each ethnic group, however, the manifestation of these barriers may differ according to the cultural and immigrant heritage (Table 1). For example, a deficiency in English may be less of a barrier for Japanese-Americans, who immigrated earlier and are more assimilated into American society, than for KAs who are primarily first generation immigrants. Therefore, the need for bilingual advocates for KA psychiatric patients may be greater than that for Japanese-American bilinguals. Also, because of the sheer size and geographic concentration of their ethnic population, culturally appropriate health services may be more readily available for Chinese Americans than for KAs, especially in San Francisco or New York City. Other culturally specific barriers among Koreans may include an overvalued belief of the heritable nature of mental disorders and a stoic sense of self-reliance in dealing with emotional problems. The hereditary nature of mental disorders has been traditionally and widely accepted among the Korean general population but is perceived as a shameful inherited trait in a family. For example, it can severely hinder the marital prospects of young men and women of suitable age and often persists as a well-kept family secret. This general reluctance of family members to seek help from outside sources for what is perceived to be a family secret is one of the biggest barriers. This more-or-less culturally-specific barrier requires equally specific solutions that are tailored to the tradition and structure of this ethnic community.

Another underappreciated, culturally-specific barrier is the atypical presentation of mental disorders among KAs. For example, Hwa-Byung (HB) or "anger syndrome" is a neurasthenia-like Korean culture-bound syndrome listed in DSM-IV. Attributed to suppression of anger, symptoms of HB include insomnia, fatigue, panic, fear of impending death, dysphoric affect, indigestion, anorexia, dyspnea, palpitations, generalized aches and pains, and feelings of a mass in the epigastrum.^26^ Because of its recurrent and specific symptom patterns and pathogenesis that may not be linked to a particular diagnostic mental disorder category, HB has been categorized as a culture-bound syndrome in DSM-IV. Epidemiological studies have suggested an HB prevalence of 4.2% to 5.0% in the general population in Korea.^27^-^29^ In the US, Lin and coworkers examined 109 KA community subjects and reported that 12% of the subjects suffered from HB.^30^ Culture-bound syndromes like HB, and generally dissimilar presentation of mental disorders, make diagnosis challenging for both primary care physicians and psychiatrists, and presents a barrier to health care access for KAs with mental problems that do not fit the conventional diagnostic criteria.

## The Korean Church and Korean Americans

From the earliest days of Korean immigration to the US, Christianity has been a focal point of the KA community. The earliest wave of Korean immigrants came to Hawaii to work on the sugar plantations in 1903 in order to fill the labor shortage triggered by the Chinese Exclusion Act of 1898. Numbering approximately 7,000, many of these early KA immigrants were mobilized for the journey by the Protestant churches in Korea, and 40% were Christians.^31^ The tendency for Korean immigrants to build a church wherever they lived continued throughout their immigration history. A conservative estimate in 1995 indicated that there are more than 3,500 KA churches, approximately one Korean immigrant church for every 300 KAs.^32^ Certainly, a popular quip among KAs seems to ring true: "When two Koreans meet, they establish a church." Kim's study on Asian-Americans in the Chicago area revealed that the church participation of Korean immigrants (71%) was greater than that in their Chinese sample (32%) or Japanese sample (28%).^33^

The Korean church is the single most important organizing force for the immigrant community, given its geographic dispersion. The vast majority of KAs gather together at least once a week to attend a service in a church where their children are taught to speak and write Korean and the elderly engage in variety of educational and cultural activities. The central nature of the Korean churches as the focus of Korean social life can be glimpsed in the Presbyterian Panel Study and Racial Ethnic Presbyterian Panel Studies by the Research Center of the Presbyterian Church (US). In this a mail survey of 1,900 African Americans, 1,072 Hispanics, and 1,355 Koreans, four-fifths of Koreans (78%) reported that they attended their congregation's Sunday worship every week, as compared to 34% of African Americans, 49% of Hispanics, and 28% of Caucasians.^34^ In fact, KA Christians quite commonly attend church three or more times a week; they not only regularly attend the Sunday morning service, but also the Sunday afternoon service, the Wednesday evening service, the Saturday morning prayer service, and so on. Given that so many Korean immigrants are engaged in small businesses with extended working hours that preclude time for socialization and recreation on weekdays, church activities provide the only opportunity for socializing with their peers and engaging in family activities for the vast majority of KAs.

## The Role of the Clergy in Promoting Mental Health Among Korean Americans

Because of the central role played by Korean churches in meeting social and cultural needs in the daily lives of KAs, members of the clergy play a very important leadership role in the KA community. The clergy of Korean churches, however, assume more than the traditional role of a spiritual leader; they are also social service providers, teachers, interpreters, and counselors for those KAs in need. In the earlier days of immigration, Korean clergy were the first to greet the anxious Korean immigrant families upon their arrival at the airport; they helped them with their luggage, found them an apartment to rent, assisted them with their job search, and even enrolled their children in the appropriate schools.

While no specific data exist, it can be safely assumed that more KAs with emotional and mental problems seek help from clergy than from their primary care physicians. As the majority of KAs attend church and the clergy are likely to serve as the first point of contact for the emotionally troubled in need of psychiatric treatment, these church leaders are more likely to act as the "gate keepers" to mental health services than are the primarily care physicians. In addition to their easy accessibility, having clergy as the "gatekeeper" provides other advantages, in terms of allowing KAs to initially avoid the stigma of seeking help from a mental health professions and offers them access to a culturally sensitive professional who is experienced in dealing with a variety of spiritual and life difficulties.

However, potential pitfalls exist for KAs who rely solely on the clergy for help with mental and emotional problems. While most clergymen are experienced in helping people with spiritual issues and life stressors (e.g. bereavement or marital discord), few are trained to recognize and deal with major mental disorders such as major depressive disorder, bipolar disorder, and schizophrenia. Even when they do recognize a mental disorder, members of the clergy may have little knowledge about available mental health service resources in the community. Also, in some cases, their clergy's spiritual conceptualization of psychiatric symptoms might delay an appropriate psychiatric intervention. Kim-Goh interviewed 50 Korean clergymen with an average duration in ministry of more than 20 years and found more than half of them conceptualized psychotic symptoms of religious delusions as primarily a religious problem rather than a psychiatric problem, and half of them reported that they had never made a referral to a mental health professional.^35^ The same study found that the Korean clergymen with a psychological conceptualization of psychiatric symptoms were far more likely to make a referral for mental health treatment than were clergy with religious conceptualization. These findings suggest that conducting outreach programs or educational workshops for Korean clergy on the psychological and medical aspects of psychiatric disorders may enhance their sensitivity and increase the referral rate to the mental health services.

## The Association of Korean American Psychiatrists and Community Workshops on Psychiatric Disorders for Korean Clergy

The Association of Korean American Psychiatrists (AKAP) was founded in 1979 primarily as a social organization for KA psychiatrists who were first-generation immigrants. Some like-minded members of this national voluntary organization became interested in helping fellow KAs in their psychiatric service needs. As a result, AKAP has re-considered and re-defined its organizational goals to focus its attention on the unmet need for mental health services within the KA community. This group concluded that direct outreach efforts to combat the stigma of mental disorders and to improve mental health service in general KA population would not be efficacious, in part because the KA population is too geographically scattered to be covered effectively by the limited AKAP membership. However, the group recognized that Korean clergy, as influential leaders of the KA community and potential "gate keepers" of mental health services were a viable, identifiable target group for the AKAP's efforts to achieve community-wide outreach to improve access to mental health service among KAs. The AKAP has organized a number of community workshops on the etiology and treatment of psychiatric disorders for the Korean clergy, the first of which was held in Chicago at a local Korean church in 2000. Approximately 30 clergyman and 10 KA psychiatrists attended the workshop, which covered by the local Korean TV station and newspaper. The success of this workshop led to wider audiences in San Francisco in 2003, New York City in 2004, and Atlanta in 2005.

**FIGURE 1:**
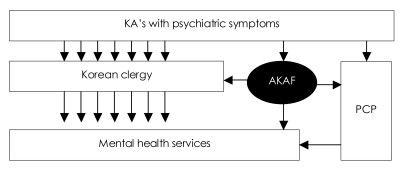
Proposed model of mental health service delivery for KA's. KA: Korean American, AKAP: Association of Korean American Psychiatrists, PCP: primary care physicians.

This outreach activity targeting Korean clergy has transformed the role of AKAP from a primarily sociopolitical organization to a service organization that seeks to improve the state of mental health for KAs. Figure 1 displays the currently proposed model for improving access to mental health care for KAs. As implied in the diagram, for the vast majority of the KAs with psychiatric symptoms, Korean clergy will continue to be the first point of contact, while the primary care physicians will continue to serve as gate keeper for a small fraction of them. The AKAP is strategically positioned to make an impact by facilitating and supporting "gate keepers" to the appropriate mental health services for the KAs in need. Thus far, much of its effort has been directed toward opening create a dialogue with the Korean clergy in the major metropolitan areas and explaining the medical model of psychiatric disorders. In addition, AKAP has established the Luke and Grace Kim Research Fund and initiated a study to assess and the Korean clergy's conceptualization of psychiatric disorders and their attitudes toward mental health service in comparison to those held by the practicing Korean primary care physicians and psychiatrists. The findings from this ongoing study will help improve the format and content of future workshops for Korean clergy.

Thus far, AKAP-sponsored symposia and workshops targeting Korean clergy members have increased awareness of mental disorders in KA communities and have led to an ongoing dialogue with Korean clergy. However, a glaring shortcoming of the AKAP-sponsored program to date has been the paucity of efforts to link the Korean clergy to local mental health services. Because of a lack of available Korean mental health professionals, identification by Korean clergy of KAs with psychiatric service needs could lead to a dead end. A feasible system of referral to the appropriate and local mental health services has to be provided to the Korean clergy as part of the effort to develop an ongoing partnership to meet the psychiatric needs of KAs. Identifying culturally sensitive, local mental health professionals and increasing their availability would be the ideal solution, yet this goal is currently unrealistic because of sheer lack of available financial resources and personnel. Instead more efforts should be put into linking the clergy to the existing Korean service organizations with local chapters such as the Korean Family Counseling Service Center, the Korean Resource Center, and the Korean Women Service Center, to create an advocacy resource to ensure smooth flow of care for the KAs in need of psychiatric care. The availability of a feasible referral and advocacy system for the Korean clergy to utilize in times of psychiatric service need would facilitate meeting the needs of mental health service needs of KAs.

## Conclusion

Despite the rapid increase of the KA population in the US, mental health service utilization by KAs has lagged behind. Barriers to mental health service access among KAs are similar to those in other Asian immigrant communities, but the manifestations of these common barriers in KA community reflect its reflect its unique heritage and culture. In addition to being religious leaders of the KA community, Korean clergy are influential in daily lives of the vast majority of KAs who are Christians. By working with the clergy to improve the mental health service access for KAs in need of proper psychiatric care, a small voluntary organization such as the AKAP could provide invaluable assistance in removing the barriers to mental health services for KAs.

## Figures and Tables

**TABLE 1:**
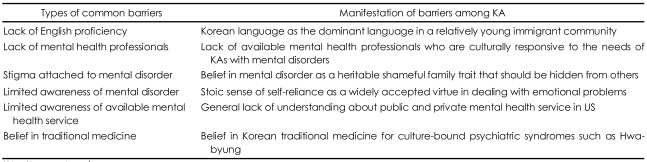
Types and manifestations of barriers to mental health services among KAs

KAs: Korean Americans
